# Ola Mau I Ka Hula: An Intervention Approach Strategy

**DOI:** 10.1002/alz.091020

**Published:** 2025-01-09

**Authors:** Ke'alohilani Worthington Antonio

**Affiliations:** ^1^ University of Hawaiʻi, Department of Native Hawaiian Health, Honolulu, HI USA

## Abstract

The prevalence of Alzheimer’s disease and related dementias (ADRD) is rising in Hawai`i, where it is projected that 10% of people aged 65+ will be diagnosed with ADRD by 2025. Nationwide, Native Hawaiians and Pacific Islanders (NHPIs) are more likely to have early‐onset ADRD than Whites, Asians, and African Americans. Contributing factors to this are that NHPIs have a higher prevalence of hypertension, obesity, diabetes, and dyslipidemia than other US racial and ethnic groups, which increase the likelihood of ADRD.

Ola Mau I Ka Hula is an intervention program being implemented by the Natives Engaged in Alzheimerʻs Research (NEAR): IKE Kupuna Project. This program is the successor to Ola Hou I Ka Hula, the intervention program for the KāHOLO Project that focused on cardiovascular risk factors in Native Hawaiian populations. This culturally‐grounded approach centers on the use of hula and lifestyle interventions to mitigate the vascular risk factors that increase the risk for mild cognitive impairment (MCI) and subjective cognitive impariment (SCI), and subsequently also ADRD. Factors such as socialization, physical activity, and learning a language are known to improve risk for ADRD. By design, hula targets multiple risk factors for ADRD and incorporates these risk reducers with a cultural twist.

This approach focuses on building on previous clinical trials and interweaving those interventions, which includes the PILI Lifestyle Program (PLP) and the KāHOLO Project. The melding the culturally‐informed components of traditional hula classes, lifestyle intervention classwork, and cultural classes teaching traditional crafts and knowledge. Community members and hula experts were consulted to adapt and strengthen the intervention strategy and the various curriculum components. The intervention was further adjusted depending on each partnering communityʻs available resources in order to ensure the sustainability of the program.

